# De-novo NAD^+^ synthesis regulates SIRT1-FOXO1 apoptotic pathway in response to NQO1 substrates in lung cancer cells

**DOI:** 10.18632/oncotarget.11526

**Published:** 2016-08-23

**Authors:** Huiying Liu, Rong Xing, Xuefang Cheng, Qingran Li, Fang Liu, Hui Ye, Min Zhao, Hong Wang, Guangji Wang, Haiping Hao

**Affiliations:** ^1^ State Key Laboratory of Natural Medicines, Key Laboratory of Drug Metabolism and Pharmacokinetics, China Pharmaceutical University, Nanjing 210009, China; ^2^ Department of Physiology and Pathophysiology, Basic Medical College of Peking University, Beijing 100191, China; ^3^ Department of Pharmacy, The First Affiliated Hospital of Bengbu Medical College, Bengbu, Anhui 233004, China

**Keywords:** NQO1, FOXO1, SIRT1, NAD^+^, LAT1

## Abstract

Tryptophan metabolism is essential in diverse kinds of tumors via regulating tumor immunology. However, the direct role of tryptophan metabolism and its signaling pathway in cancer cells remain largely elusive. Here, we establish a mechanistic link from L-type amino acid transporter 1 (LAT1) mediated transport of tryptophan and the subsequent de-novo NAD^+^ synthesis to SIRT1-FOXO1 regulated apoptotic signaling in A549 cells in response to NQO1 activation. In response to NQO1 activation, SIRT1 is repressed leading to the increased cellular accumulation of acetylated FOXO1 that transcriptionally activates apoptotic signaling. Decreased uptake of tryptophan due to the downregulation of LAT1 coordinates with PARP-1 hyperactivation to induce rapid depletion of NAD^+^ pool. Particularly, the LAT1-NAD^+^-SIRT1 signaling is activated in tumor tissues of patients with non-small cell lung cancer. Because NQO1 activation is characterized with oxidative challenge induced DNA damage, these results suggest that LAT1 and de-novo NAD^+^ synthesis in NSCLC cells may play essential roles in sensing excessive oxidative stress.

## INTRODUCTION

NAD(P)H: quinone oxidoreductase 1 (NQO1), a flavin protease enzyme prevalent in most eukaryotic cells, has attracted considerable attention because of important roles in cancer chemoprevention and oncotherapy [1]. NQO1 is overexpressed in a variety of tumors compared to normal adjacent tissues [2], the characteristic of which confers an optimal window to develop anti-tumor agents with high selectivity towards tumor but sparing normal tissues [3]. The FDA of U.S.A has approved a panel of phase II clinical trials for testing the chemotherapeutic efficacies of β-lapachone (β-lap), an NQO1 targeting agent, either alone or in combinatory use with other marketed chemotherapeutic drugs. We have recently identified that Tanshinone IIA (TSA), a natural compound contained in Danshen, elicits its anti-tumor efficacy via targeting NQO1 [4, 5]. It has been well verified that such NQO1 targeting agents induce cell death largely due to bioreduction triggered futile redox cycle [6]. However, the specific mechanism of how cancer cells sense NQO1-triggered redox conditions and thereafter initiating apoptotic and/or necroptotic cell death remains largely elusive. This represents a major bottleneck in successful development of NQO1 activation agents to chemotherapeutic drugs.

Previous studies showed that NQO1 bioactive agents induce p53-independent cell death [7], indicating p53 unlikely the sensor of NQO1-initiated apoptotic signals. Forkhead box O (FOXO) genes, a family of transcriptional factors containing four isoforms FOXO1, FOXO3a, FOXO4, and FOXO6 in human, are involved in diverse intracellular signaling pathways parallel to p53 [8, 9]. Moreover, FOXOs have been identified as critical sensors of cellular redox status via various mechanisms. In response to oxidative challenge, FOXOs are activated and translocate to nuclear to transcriptionally upregulate their downstream target genes such as catalase [10] and manganese superoxide dismutase (MnSOD) [11], which are responsive for excessive ROS clearance. However, under high levels of oxidative stress, inappropriately prolonged FOXOs activation induce a programmed cell death by activating apoptosis related genes such as Bcl-2 family member BIM, death cytokines Fas ligand, TRAIL, and Bcl-6 [12].

The fine-tuned mechanisms of how FOXOs coordinate with other cellular machineries to selectively upregulate detoxification genes to combat oxidative stress or proapoptotic genes to induce cell death remain largely obscure. FOXO proteins can be acetylated by histone acetyltransferase (HATs) such as p300, the cyclic-AMP responsive element binding (CREB)-binding protein (CBP) and CBP-associated factor (P/CAF), while be deacetylated by silent mating type information regulation 2 homolog 1 (SIRT1) and other histone deacetylases (HDACs). It seems that the acetylation modification is important for FOXOs to activate downstream pro-apoptotic signals. For example, overexpression of SIRT1, an NAD^+^ dependent deacetylase, inhibits both FOXO3a and FOXO1 induced apoptotic cell death while increases their ability in inducing cell cycle arrest and resistance to oxidative stress [13, 14]. Moreover, oxidative stress can also suppress SIRT1 activity by directly decreasing its protein levels, inhibiting its enzymatic activity by reducing the cellular level of NAD^+^, rendering the protein for proteasomal degradation [15]. NAD^+^ serves as a critical coenzyme in fuel reduction-oxidation reactions, also as a cosubstrate for enzymes such as the sirtuins and poly (adenosine diphosphate-ribose) polymerases (PARP). Cellular NAD^+^ concentrations change during aging [16], inflammation [17] and other diseases. De novo synthesis of NAD^+^ from tryptophan may play an important part in cancer cells resistant to oxidative stress [18] and is increasingly being recognized as an important microenvironmental factor that suppresses antitumor immune responses [19]. Thus, NAD^+^-SIRT1-FOXOs signaling pathway is proposed playing pivotal roles in sensing cellular redox status under various physiological and pathological settings. Taking together, we hypothesized that NAD^+^-SIRT1-FOXOs signaling may be an important sensor of NQO1 triggered oxidative stress initiating apoptotic signaling pathways. In this study, we showed that the nuclear translocation of acetylated FOXO1 in response to NQO1 activation-triggered oxidative stress elicits apoptotic cell death. The accumulation of acetylated FOXO1 is attributed to the decreased SIRT1 activity caused by both the transcriptional downregulation and the depletion of NAD^+^ pool. Furthermore, PARP-1 hyperactivation consumes large amount of NAD^+^, rendering cells relying on NAD^+^ de-novo synthesis from tryptophan. However, tryptophan transmembrane transport was blocked due to L-type amino acid transporter 1 (LAT1) inhibition, leading to an irreversible NAD^+^ depletion and subsequent activation of SIRT1-FOXO1 apoptotic signaling.

## RESULTS

### NQO1 substrates activates FOXO1 signaling

**Figure 1:**
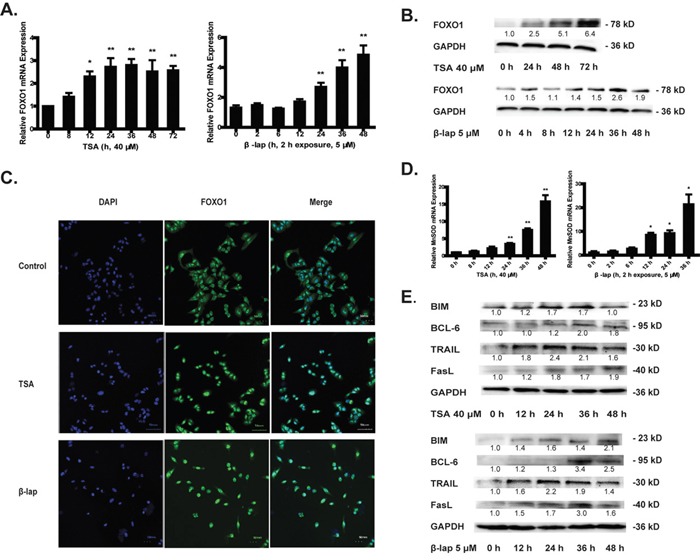
NQO1 substrates TSA and β-lap induce FOXO1 activation A549 cells were treated with 40 μM of TSA or 5 μM of β-lap for 2 h and harvested at indicated time. The mRNA level **A.** and protein level **B.** of FOXO1. FOXO1 protein translocation from cytoplasm into nucleus was observed by confocal microscope **C.** The mRNA level of MnSOD **D.** The protein levels of BIM, BCL-6, TRAIL and FasL **E.** Data are shown as mean ± SEM of three independent experiments (*P<0.05, **P<0.01, TSA or β-lap treatment compared with control group).

TSA [4] and β-lap [20] can both trigger NQO1 bioactivation and induce apoptosis of cancer cells. It is important to note that a short-term of β-lap exposure (2-8 h) triggers NQO1-mediated cancer cell apoptosis which can be eliminated by NQO1 inhibitor dicoumarol, while long-term exposure of β-lap (>12 h) induces nonselective damage to DNA or microtubules [21]. In this study, we thus chose an appropriate exposure of β-lap for 2 h and the cells were then treated for indicated time without β-lap. To determine whether FOXOs were involved in sensing NQO1 activation-triggered cellular redox conditions, we first tested the change of mRNA and protein levels of FOXOs upon NQO1 activation. Mammalian FOXO proteins include FOXO1, FOXO3, FOXO4, and FOXO6. Since FOXO6 is dominantly expressed in brain [22], we examined the changes of FOXO1, FOXO3 and FOXO4 levels in response to NQO1 activation by TSA and β-lap in NQO1-highly expressed A549 cells. Cells were exposed to 40 μM of TSA for series of time, or 5 μM of β-lap for 2 h and then cultured in fresh medium for series of time. A significant and sustaining FOXO1 upregulation of both mRNA and protein levels was induced by both agents (Figure 1A, 1B), while no significant changes were observed for FOXO3 and FOXO4 (Supplementary Figure S1A). FOXO1 upregulation was reversed by NQO1 specific inhibitor dicoumarol (Supplementary Figure S2A, S2B) and NQO1 siRNA pretreatment (Supplementary Figure S2C, S2D, S2E) in A549 cells, and was induced in NQO1^+/+^-H596 cells but not in NQO1^−/−^-H596 cells (Supplementary Figure S2F), demonstrating a NQO1 on-target effect by both agents. Given that FOXOs should translocate into nucleus to transcriptionally activate downstream targets, nuclear accumulation of FOXO proteins was visualized by confocal microscopy. TSA and β-lap challenge induced significant nuclear accumulation of FOXO1 (Figure 1C) but not FOXO3 proteins (Supplementary Figure S1B). All these results support that FOXO1, but not other FOXO isoforms, is involved in sensing NQO1 activation triggered oxidative stress in A549 cells.

FOXO1 is implicated in many fundamental cellular processes through the transcriptional regulation of a wide array of targeting genes, and the regulation of its downstream targeting genes serving as real executants may thus determine cell fates when FOXO1 activity is enforced [8, 12]. In response to both TSA and β-lap challenge, the typical FOXO1 targeting gene MnSOD which is involved in ROS clearance was adaptively upregulated (Figure 1D) while catalase was undetectable in A549 cells(data not shown). Moreover, the FOXO1 downstream proapoptotic proteins including death cytokines (FasL, TRAIL), apoptotic Bcl-2 family member (BIM) and transcriptional repressor (BCL-6) of anti-apoptotic BCL-xl were all upregulated in a time-dependent manner reaching the highest level at 36 h after NQO1 substrates treatment (Figure 1E). These results suggest that FOXO1 transcriptional activity was activated in response to NQO1 triggered oxidative stress.

### NQO1-initated apoptotic cell death is FOXO1-dependent

**Figure 2:**
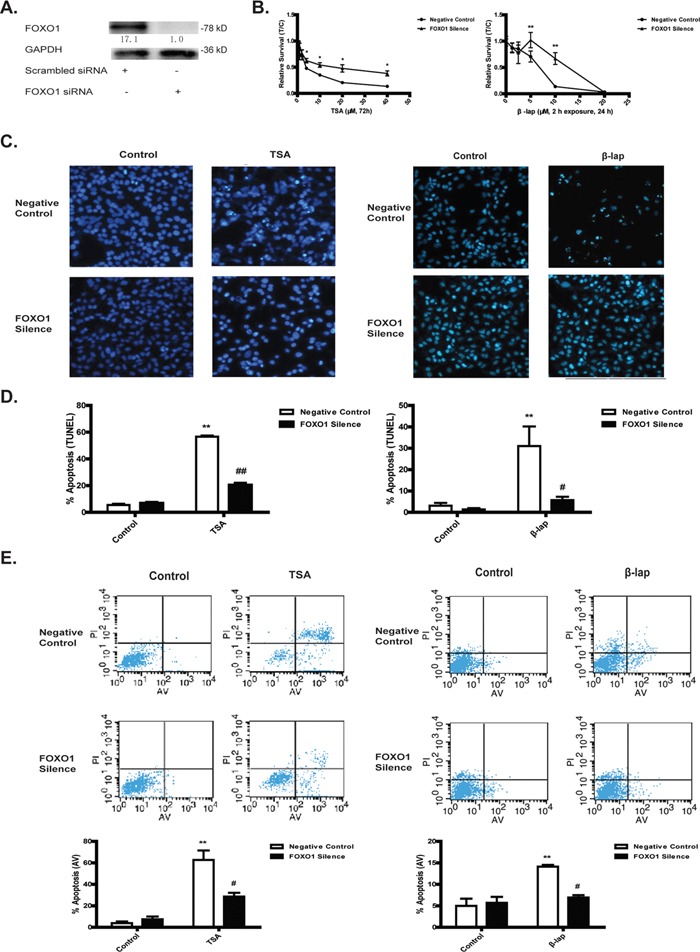
NQO1 substrates-initated apoptotic cell death is FOXO1-dependent A549 cells were treated with FOXO1 siRNA or scrambled siRNA for 24 h. Cells pretreated with siRNA were then treated with TSA (40 μM, 72 h) or β-lap (5 μM, 2 h exposure, 24 h). Silencing efficacy **A.** Cytotoxicity test by MTT **B.** DNA damage assessed by Hoechst 33342 staining **C.** Cell apoptosis assessed by TUNEL assay **D.** and Annexin V-FITC/PI assay **E.** Data are shown as mean ± SEM of three independent experiments (**P<0.01, TSA or β-lap treatment compared with control group; #P<0.05, ## P<0.01, FOXO1 siRNA pretreatment compared with scrambled siRNA pretreatment).

Because NQO1 activation promotes the transcriptional activity of FOXO1 and thereby upreguating its downstream proapoptotic genes, we wondered whether FOXO1 determined NQO1 activation-induced cell death. To this end, TSA and β-lap cytotoxicity were tested in A549 cells pretreated with FOXO1 siRNA or scrambled siRNA. FOXO1 siRNA efficacy was validated by western blot assay (Figure 2A). Knockdown of FOXO1 by siRNA dramatically reversed the cytotoxicity of TSA at all tested concentrations, and reversed the cytotoxicity of β-lap at relatively low concentrations (Figure 2B) but not at high concentrations (20 μM), which is possibly due to NQO1-nonspecific cytotoxicity of β-lap at high concentrations [23]. Hoechst 33342 staining tests proved that TSA and β-lap led to nuclear pycnosis and apoptotic body formation of A549 cells, suggesting a characteristic apoptosis in this condition, which was also reversed by FOXO1 siRNA pretreatment (Figure 2C). TUNEL assay and Annexin V-FITC/PI apoptotic assay performed with Flow Cytometry analysis further demonstrated that FOXO1 knockdown significantly rescued TSA and β-lap induced apoptosis of A549 cells (Figure 2D, 2E). In addition, NQO1 activation induced apoptotic cell death was associated with dramatic ATP loss, while FOXO1 knockdown largely abrogated this effect (Supplementary Figure S3). Together, these results clearly indicate that FOXO1 plays a pivotal role in NQO1 activation-triggered apoptotic cell death.

### SIRT1 determines NQO1 mediated cell apoptosis

**Figure 3:**
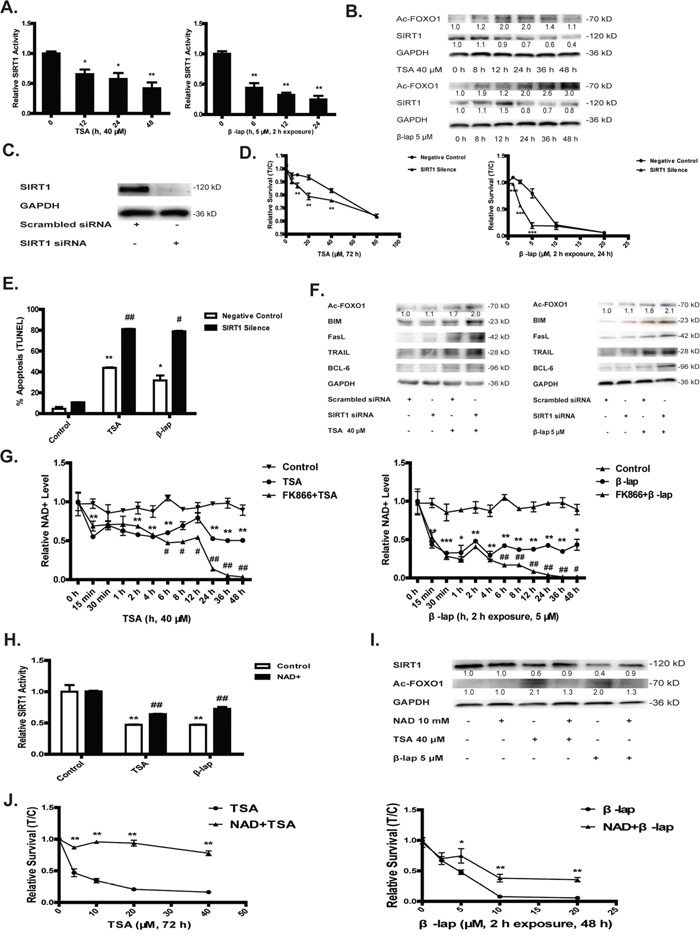
SIRT1 determines NQO1 mediated cell apoptosis The enzymatic activity **A.** and protein levels **B.** of SIRT1 and Ac-FOXO1. A549 cells were treated with SIRT1 or scrambled siRNA for 24 h and silencing efficacy was determined by western blot assay **C.** Cells pretreated with SIRT1 siRNA was then treated with TSA, 40 μM, 48 h or β-lap, 5 μM, 2 h exposure, 24 h. Cytotoxicity **D.** and apoptosis test **E.** The protein levels of Ac-FOXO1 and the downstream apoptotic proteins were estimated **F.** Time-course study of NAD^+^ levels with or without pretreatment of NAMPT inhibitor FK866 10 nM for 1 h **G.** 10 mM of NAD^+^ supplementation for 1 h reversed TSA or β-lap induced SIRT1 inhibition and Ac-FOXO1 accumulation **H, I.** as well as cytotoxicity **J.** Data are shown as mean ± SEM of three independent experiments (*P<0.05, **P<0.01, TSA or β-lap treatment compared with control group; #P<0.05, ##P<0.01, SIRT1 siRNA treatment compared with scrambled siRNA treatment).

Transcriptional activity of FOXO1 is regulated by various forms of post-translational modification, such as phosphorylation, monoubiquitylation and acetylation. Among these post-translational modifications, it is believed that acetylation is closely associated with the transcriptional activation of apoptotic signaling by FOXO1 in response to oxidative challenge, which is negatively regulated by sirtuin-catalyzed deacetylation. From this consideration, we asked whether SIRT1 activity could be an important determinant in NQO1 activation-induced apoptotic cell death. As expected, NQO1 activation by both TSA and β-lap significantly decreased the enzyme activity (Figure 3A), the mRNA (Supplementary Figure S4) and protein levels of SIRT1 (Figure 3B), leading to an accumulation of Ac-FOXO1 (Figure 3B). Though SIRT1 mRNA was observed to be raised during the first 8 h treatment of TSA, which may be explained by an adaptive cellular response to oxidative stress, a subsequent repression of SIRT1 mRNA occurred in the apoptosis progress (Supplementary Figure S4A). NQO1 inhibitor DIC largely reversed both agents induced SIRT1 repression and Ac-FOXO1 accumulation (Supplementary Figure S5), supporting an on-target effect of both agents in the regulation of SIRT1 signaling. The pretreatment of A549 cells with SIRT1 siRNA (Figure 3C) significantly sensitized their susceptibility to NQO1 activation by both agents induced cytotoxicity and apoptosis, especially at relatively low concentration (Figure 3D, 3E). SIRT1 knockdown also increased the accumulation of acetylated FOXO1 and the protein levels of its downstream proapoptotic genes (Figure 3F). These data suggest that SIRT1 may act as an upstream component of FOXO1 in sensing NQO1 activation-triggered oxidative stress and initiating apoptotic cascade.

### NAD^+^ pool depletion initiates NQO1-activation induced apoptosis

It is well known that SIRT1 is NAD^+^-dependent deacetylase. Upon SIRT1 activation, NAD^+^ is utilized as a substrate to receive the acetyl group producing NAM serving as SIRT1 inhibitor. Thus, NAD^+^/NAM cycling represents an important feedback mechanism in the regulation of SIRT1 activity. Although it was previously reported that β-lap induced cell death is characterized with NAD^+^ depletion, it is unknown whether NAD^+^ depletion is an initiating factor in the cascade of NQO1 activation induced cell death. Here, we demonstrated that in response to NQO1 activation by both TSA and β-lap, the NAD^+^ pool was rapidly depleted within 2 hrs (Figure 3G). Of interest, NAD^+^ replenishment largely reversed NQO1 activation-induced SIRT1 repression, Ac-FOXO1 accumulation and cytotoxicity in A549 cells (Figure 3H, 3I, 3J), supporting that NAD^+^ depletion is a vital mechanism in initiating cell death upon NQO1 activation.

The results collected above suggest that NAD^+^-SIRT1-FOXO1 signaling is a crucial determinant in NQO1 activation-triggered cell death. To validate that this signaling is initiated from the sense of oxidative stress, we determined the effects of ROS scavenger N-Acetylcysteine (NAC) in the regulation of this signaling pathway. In line with previous reports, NAC treatment largely abolished NQO1 activation induced cytotoxicity (Supplementary Figure S6D). Besides, 1 h of NAC pretreatment recovered both the mRNA levels and enzyme activity of SIRT1 and largely reversed NQO1 activation-induced Ac-FOXO1 accumulation (Supplementary Figure S6). Together, these results indicate that NAD^+^-SIRT1-FOXO1 signaling activates the downstream apoptotic cascade through the sense of NQO1 activation induced redox status.

### Salvage synthesis of NAD^+^ is adaptively activated

**Figure 4:**
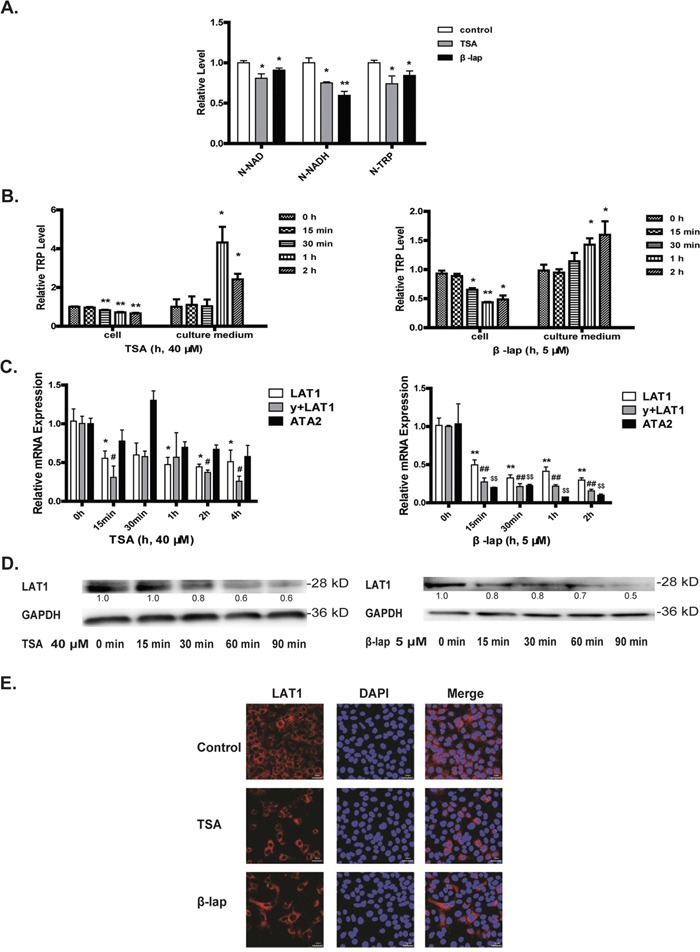
Decreased tryptophan transport explains NAD^+^ depletion A549 cells were treated with TSA (40 μM) or β-lap (5 μM, 2 h withdraw). The intracellular N-NAD, N-NADH, and N-TRP in A549 cells supplemented with isotope labeled tryptophan and treated with drugs for 4 h **A.** The levels of tryptophan in the cells and culture medium were detected by LC-MS^n^ **B.** The mRNA levels of several tryptophan transporters (LAT1, y+LAT1, ATA2) **C.** The protein levels of LAT1 assessed by western blot assays **D.** and confocal microscope with drug exposure of 1 h **E.** (*P<0.05, **P<0.01, TSA or β-lap treatment compared with control group; #P<0.05, ##P<0.01, y+LAT1 mRNA expression with TSA or β-lap treatment compared with control group; $P<0.05, $$P<0.01, ATA2 mRNA expression with TSA or β-lap treatment compared with control group).

Since NQO1 activation induced NAD^+^ depletion is an early and fast process and it is suggested that NAD^+^ is an upstream component in SIRT1-FOXO1 signaling, we sought to verify the mechanisms underlying the fast depletion of NAD^+^ upon NQO1 activation. Previous reports suggested that β-lap induced cell death is due to PARP-1 activation utilizing NAD^+^ as a substrate to produce NAM and polyADP-ribose. We tested PARP-1 activation in NQO1 substrates-induced oxidative stress, finding an elevated PAR expression (Supplementary Figure S7). The pretreatment of PARP-1 inhibitor such as DPQ and AZD2291 could largely combat against NQO1 substrates induced depletion of NAD^+^, downregulation of the expression and enzyme activity of SIRT1, accumulation of Ac-FOXO1, and cell apoptosis (Supplementary Figure S8). These results confirmed that PARP-1 activation is indeed an important factor in the depletion of NAD^+^ and thereafter the activation of SIRT1-FOXO1 apoptotic pathway. PARP-1 activation mediates the metabolism of NAD^+^ to NAM and thus is reasonable to expect NAM should be increased upon NQO1 activation. Surprisingly, the intracellular level of NAM was also decreased in a time-dependent manner, failed to prove PARP-1 determining NAD^+^ pool depletion. Because NAM can be reversely metabolized to NAD^+^ mediated by NAMPT and NMNAT in the salvage synthesis of NAD^+^, we sought to determine whether the salvage pathway of NAD^+^ was altered upon NQO1 activation. The expression of both enzymes Nicotinamide phosphoribosyltransferase (NAMPT) and Nicotinamide mononucleotide adenylyltransferase (NMNAT) was significantly upregulated upon NQO1 activation (Supplementary Figure S10). Besides, treatment of FK866, an NAD^+^ salvage synthesis key enzyme NAMPT inhibitor, further decreased NAD^+^ level 2 h after treatment of NQO1 substrates and finally induced a complete depletion of NAD^+^ pool (Figure 3G). These results indicate that the salvage synthetic pathway is compensatory activated to combat the complete depletion of NAD^+^ pool and maintain the cellular NAD^+^ at a stable but relatively low level in response to NQO1 activation. However, it remained unclear why the entire NAD^+^ pool including NAM is drastically reduced in response to NQO1 activation. Thereafter, we questioned whether the NAD^+^ de novo synthesis pathway from tryptophan was compromised upon NQO1 activation. To this end, we performed a comprehensive analysis of all the intermediates and products involved in NAD^+^ de-novo synthesis. NQO1 activation by both TSA and β-lap induced a significant intracellular loss of all the intermediates (Figure 4A, Supplementary Figure S9A) and the precursor tryptophan (Figure 4B), hinting to a compromised de-novo synthesis of NAD^+^. We next determined the time course change of major enzymes (IDO, TDO, QPRT and KMO) which are involved in de-novo synthesis of NAD^+^. Unexpectedly, all these NAD^+^ synthetic enzymes underwent a compensatory increase or at least unchanged except for KMO (Supplementary Figure S10), suggesting that the compromised de-novo synthesis of NAD^+^ is unlikely due to the downregulation of enzymes.

### Decreased cellular uptake of tryptophan explains NAD^+^ depletion

De-novo synthesis of NAD^+^ initiates from tryptophan, the cellular uptake of which is an active process mediated by LAT1. We hypothesized that the tryptophan transport might be repressed in response to NQO1 activation. To test this hypothesis, we applied a stable isotope ^15^N2 labeled tryptophan used as NAD^+^ synthetic source to trace the NAD^+^ synthetic pathway. NQO1 activation by both TSA and β-lap induced a significant decrease of cellular uptake of ^15^N2-tryptophan and in particular, its downstream metabolites ^15^N-NADH and ^15^N-NAD were also significantly decreased (Figure 4A). Moreover, the monitoring of tryptophan time-course kinetics showed a gradually decreased intracellular level but an increased retention in cell culture medium upon NQO1 activation, as compared with that under basal conditions (Figure 4B). All these results strongly support a repressed cellular uptake of tryptophan, the precursor for de-novo synthesis of NAD^+^. We next asked whether or not the repressed cellular uptake of tryptophan and thereafter the compromised de-novo synthesis of NAD^+^ could be an important factor in NQO1 activation induced apoptotic cell death. To this end, various kinds of NAD^+^ de novo synthetic precursors were replenished to the cells to determine NAD^+^ catabolism and the resultant cell fates. Replenishment of NAD^+^ de novo synthetic precursors such as nicotinic acid (NA), quinolinic acid (QA) or 3-hydroxykynurenine (3-HK) dramatically increased NAD^+^ levels of the cells with NQO1 activation (Supplementary Figure S9B). Moreover, the supplementation of KYN and QA (Supplementary Figure S9C) but not tryptophan (Supplementary Figure S11) significantly reversed NQO1 activation induced cytotoxicity. These results strongly support that the cells in response to NQO1 activation may shift to rely on the de-novo synthesis pathway to maintain NAD^+^ levels and that the repressed cellular uptake of tryptophan may represent an important mechanism to initiate apoptotic cell death in response to NQO1 activation.

### LAT1 determinates NAD^+^ homeostasis and SIRT1-FOXO1 signaling

**Figure 5:**
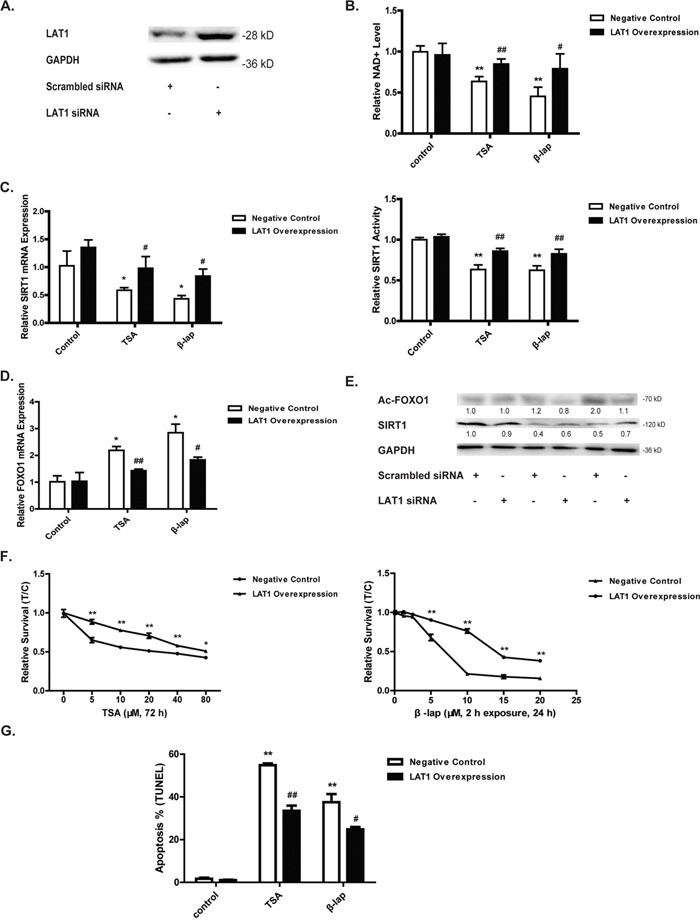
LAT1 overexpression attenuates NQO1 activation induced cell death Cells were pretreated with LAT1 plasmid for 24 h and the efficacy was evaluated by western blot **A.** The NAD^+^ level **B.** Cells were then treated with TSA (40 μM, 24 h) or β-lap (5 μM, 2 h withdraw, 12 h). The mRNA level and enzyme activity of SIRT1 **C.** The mRNA level of FOXO1 **D.** The protein levels of Ac-FOXO1 and SIRT1 **E.** Cytotoxicity **F.** and apoptosis test **G.** were performed after cells treated with TSA (40 μM, 48 h) or β-lap (5 μM, 2 h withdraw, 24 h). Data are shown as mean ± SEM of three independent experiments (*P<0.05, **P<0.01, TSA or β-lap treatment compared with control cells; #P<0.05, ##P<0.01, LAT1 plasmid treatment compared with vector treatment).

The uptake of tryptophan into cells is an active process depending on L-amino acid transporters. The overexpression of several kinds of amino acid transporters such as LAT1 in malignant tumors has been found, which may represent an intrinsic mechanism supporting the rapid proliferation of cancer cells [24]. The treatment of A549 cells with both TSA and β-lap induced a very fast and time-dependent downregulation of the LAT1, y+LAT1 and TAT2 mRNA (Figure 4C), coinciding with the cellular kinetics of tryptophan (Figure 4B). Since LAT1 is the dominant transporter for tryptophan and is overexpressed in malignant tumors to support cell growth [25], we then focused on the study of LAT1 in response to NQO1 activation. In line with the decreased mRNA levels, the protein level of LAT1 assessed by both western blot and confocal microscope was decreased significantly upon NQO1 activation by both agents (Figure 4D, 4E). LAT1 overexpression (Figure 5A) significantly reverted TSA and β-lap induced change of NAD^+^ level (Figure 5B), SIRT1 expression and activity (Figure 5C), and the expression and acetylation of FOXO1 (Figure 5D, 5E). Cytotoxicity (Figure 5F) and apoptotic effects (Figure 5G) of TSA and β-lap were also reversed by LAT1 overexpression, demonstrating the essential role of LAT1 in NQO1 substrates induced SIRT1-FOXO1 pathway and cell death. Moreover, the knockdown of LAT1 by siRNA significantly sensitized the response of A549 cells to both TSA and β-lap induced NAD^+^-SIRT1-FOXO1 pathway activation and apoptosis (Supplementary Figure S12).

### NQO1 substrates disrupt NAD^+^-SIRT1-FOXO1 pathway in vivo

**Figure 6:**
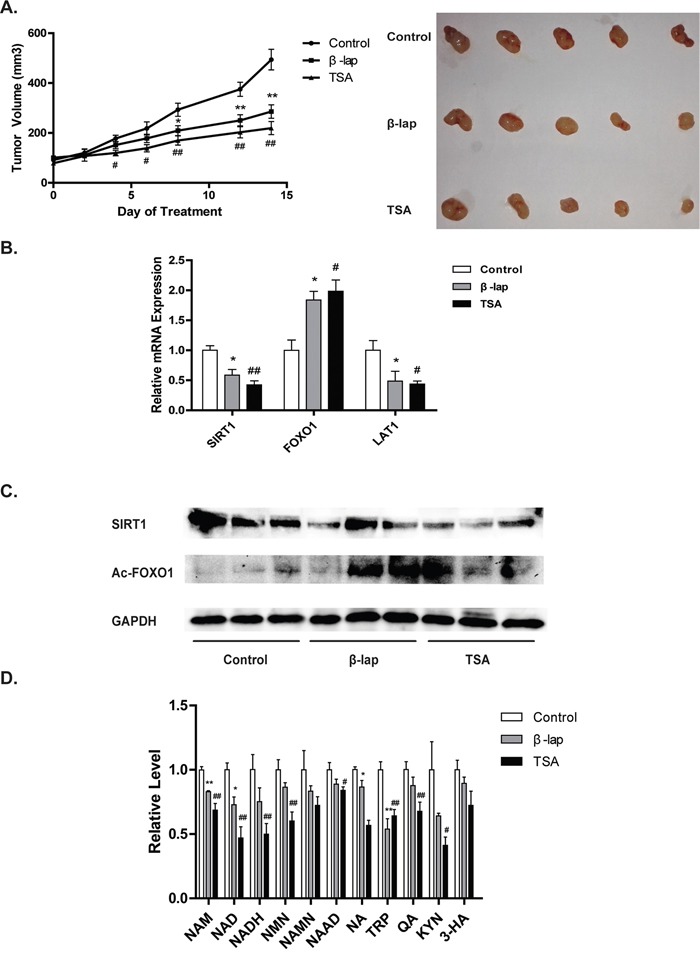
NAD^+^-SIRT1-FOXO1 pathway is activated in NQO1 substrates induced *in vivo* antitumor effect A549 tumor xenografted nude mice were administered intraperitoneally with β-lap or TSA every day for 2 weeks. Tumor volume was measured and pictured **A.** The mRNA levels of SIRT1 and FOXO1, **B.** the protein level of SIRT1 and Ac-FOXO1 were determined **C.** The level of NAD^+^ pathway intermediates were determined **D.** Data are shown as mean ± SEM of three independent experiments (*P<0.05, **P<0.01, β-lap treatment compared with vehicle; #P<0.05, ##P<0.01, TSA treatment compared with vehicle).

To confirm the importance of LAT1-NAD^+^-SIRT1-FOXO1 pathway in vivo, the antitumor efficacy of NQO1 substrates was further investigated in A549 tumor xenografts. TSA (20 mg/kg) or β-lap (30 mg/kg) significantly suppressed the tumor growth during drug treatment, evidenced by the decreased increasing rate in tumor volume (Figure 6A). After 14 days treatment, the volume of tumors was of 131.2551 mm^3^ and 162.8305 mm^3^ in TSA and β-lap treated group, respectively, both were significantly smaller than that of the control group (206.2724 mm^3^) (Figure 6A). The mRNA and protein levels of SIRT1 were decreased, while the mRNA levels of FOXO1 and the protein levels of Ac-FOXO1 were increased, in TSA or β-lap treated mice (Figure 6B, 6C). The levels of tryptophan and other metabolites involved in both the de-novo and salvage synthesis of NAD^+^ in NQO1 substrates treated mice were significantly lower than that of the control (Figure 6D). The results obtained from the tumor xenograft study further support the importance of LAT1-NAD^+^-SIRT1-FOXO1 pathway in NQO1 substrates induced antitumor effect.

### LAT1-NAD^+^-SIRT1-FOXO1 pathway is activated in patients with lung cancer

**Figure 7:**
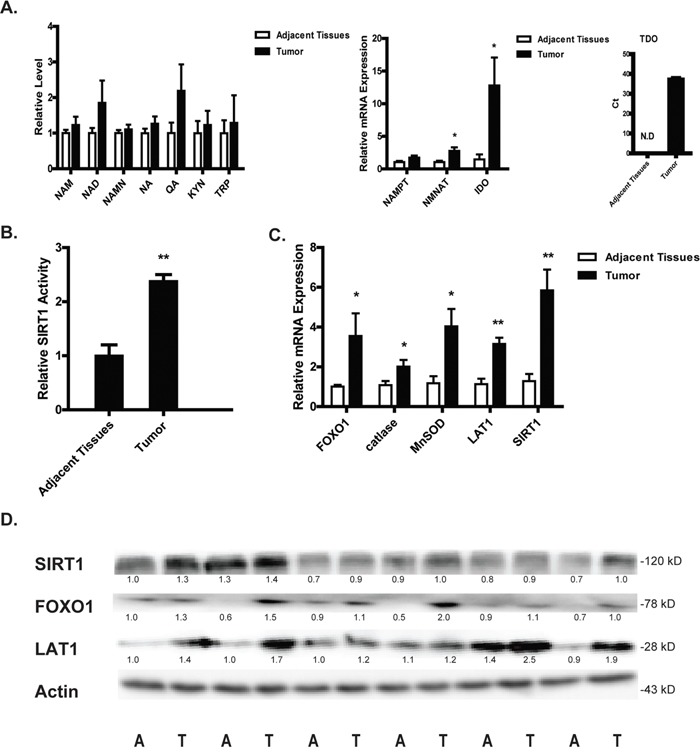
LAT1-NAD^+^-SIRT1-FOXO1 pathway is activated in patients with NSCLC The levels of NAD^+^ and its relative intermediates tumor and adjacent normal tissues from patients with NSCLC **A.** SIRT1 activity **B.** The mRNA levels of SIRT1, LAT1, FOXO1 and its downstream anti-oxidant genes **C.** The protein levels of SIRT1, FOXO1 and LAT1 **D.** Data are shown as mean ± SEM of three independent experiments (*P<0.05, **P<0.01, tumor tissues compared with adjacent tissues).

Our study indicates that NQO1 activation disrupts the de-novo synthesis of NAD^+^ from tryptophan and thereby activating the SIRT1-FOXO1 dependent apoptotic pathway. To provide a translational rationale for the use of NQO1 bioactive agents in the therapy of lung cancer, the LAT1-NAD^+^-SIRT1-FOXO1 pathway was assessed in tumor specimens in comparison with adjacent tissues from patients with non-small cell lung cancer. A slight increase of NAD^+^ and its synthetic and metabolic intermediates were found in tumor tissues in comparison with adjacent tissues (Figure 7A). Moreover, the mRNA levels of key enzymes involved in both the salvage synthesis (NAMPT and NMNAT) and de-novo synthesis (IDO and TDO) of NAD^+^ were significant higher in tumor tissues than that in the adjacent tissues (Figure 7A). Tumor tissues also showed significant high expression of LAT1. These results may suggest an increased demand of NAD^+^ synthesis and in particular the de-novo synthesis for lung cancer cells to survive and proliferate. In agreement, NAD^+^-dependent deacetylase SIRT1 displayed a higher expression and activity in tumor tissues (Figure 7B, 7C, 7D). The expression of FOXO1 and its downstream anti-oxidative stress enzymes such as catalase and MnSOD were also at a high level in tumor tissues (Figure 7C, 7D). These results hint to an adaptive activation of LAT1-NAD^+^-SIRT1-FOXO1 pathway in tumor tissues, providing a translational rationale for the use of NQO1 bioactive agents which induce apoptotic cell death via disrupting this pathway for the therapy of non-small cell lung cancer.

## DISCUSSION

Tryptophan metabolism in cancer is increasingly being recognized as an important microenvironmental factor that suppresses antitumor immune responses [26]. However, the direct roles of tryptophan metabolism in cancer cells remain largely elusive. In the current study, we demonstrated that LAT1 mediated uptake of tryptophan and subsequent de-novo synthesis of NAD^+^ is important for NSCLC cells to combat against NQO1 bioactivation-induced cell apoptosis.

In response to NQO1 activation-triggered oxidative stress, the adaptively activated salvage NAD^+^ synthesis is insufficient to compensate for the PARP-1 activation induced rapid depletion of NAD^+^, rendering the cancer cells more reliant on the de-novo NAD^+^ synthesis from tryptophan. Our study thus indicates that the increased expression of LAT1 in tumor tissues (Figure 7) might represent an adaptive mechanism for the cancer cells developing the capacity of resisting oxidative challenge via maintaining sufficient NAD^+^ levels.

LAT1 determines the transmembrane transport of many large neutral amino acids, including tryptophan. [27] LAT1 has been observed overexpressed in several solid tumors such as brain, colon, lung, liver and skin, which may represent an adaptive mechanism for cancer cells to uptake sufficient amino acids for facilitating the rapid metabolic turn-over and proliferation. [28] Several inhibitors of LAT1 have been developed and were found effective in suppressing tumor growth, cell migration and invasion [29], and increasing the sensitivity of the cancer cells to diverse therapeutic drugs such as gemcitabine, 5-FU [30], and cisplatin. [31] Beyond this well validated role of LAT1 in facilitating the uptake of sufficient nutrients, our study indicates that the overexpression of LAT1 in NSCLC cells may favor to replenish NAD^+^ via the de-novo synthesis from tryptophan, in particular when cancer cells are subject to oxidative challenge.

We demonstrated that the reduced uptake of tryptophan via the downregulation of LAT1 finally lead to the depletion of intracellular NAD^+^ pool, sensitizing the NSCLC cells to NQO1 activation-induced oxidative challenge. It was of interest to note that the expression of LAT1 was rapidly reduced upon the treatment of NQO1 substrates. Although the exact mechanism of such a rapid reduction warrants further intensive study, it is reasonable to propose that the rapid reduction of LAT1 might be related to the excessive production of oxidative stress since NQO1 substrates induced fast and continuous production of ROS. Indoleamine 2,3-dioxygenase (IDO) and tryptophan dioxygenase (TDO) are by far the most broadly studied enzymes in tryptophan-metabolism in tumor [32], providing mechanistic links from tryptophan metabolism to tumor immunosuppression. The upregulation of IDO is often associated with a poor prognosis [33] and TDO is also frequently activated in cancer [34]. However, the mechanistic link of tryptophan metabolism to tumor immunosuppression may not apply to explain the role of overexpression of IDO/TDO in cancer cells. In our study on clinical patients with NSCLC, the expression of IDO in tumor tissues is about ~12.5 fold of that in adjacent tissues, while TDO was only detected in tumor tissues (Figure 7). Moreover, the supplementation of tryptophan metabolites QA and 3-HK restored NAD^+^ levels and combated against NQO1 activation induced cell death (Supplementary Figure S9). All these evidence support that the de-novo NAD^+^ synthesis from tryptophan plays essential roles in tumor development.

Though de-novo NAD^+^ synthesis inhibition accounts for the unrecoverable loss of NAD^+^, the activation of PARP-1 contributes to the initial fast NAD^+^ depletion. PARP-1 activation is characterized with PAR polymer formation accompanying with NAD^+^ break down to NAM [35]. It is thus reasonable to predict that PARP-1 activation may result in the intracellular accumulation of NAM. However, the level of NAM is also dramatically decreased in response to NQO1 activation (Supplementary Figure S9A), which may be partially explained by the adaptively activated salvage NAD^+^ synthetic pathway utilizing NAM to promptly synthesize NAD^+^. It is of interest to note that NAMPT inhibitor significantly decreased NAD^+^ only at the latter phase (after 4 h) but not the early phase of NQO1 bioactivation (Figure 3G). These results indicate that the adaptive activation of salvage synthesis pathway helps for combating against PARP-1 mediated consumption of NAD^+^ but cannot compensate for the early depletion of NAD^+^ due to de-novo synthesis repression, thereby maintaining NAD^+^ at a stable but low level. Together, we presume that the LAT1 downregulation and the subsequent decreased de-novo NAD^+^ synthesis from tryptophan might be a pivotal mechanism explaining for NQO1 activation induced rapid depletion of NAD^+^.

NAD^+^ is not only an essential co-factor in maintaining cellular redox balance, but also a second messenger required for a number of cellular processes essential for survival. In the latter case, NAD^+^ acts as either a donor (PARP-1) or a receiver (SIRT1) to facilitating such cellular processes, accompanied with the consumption of NAD^+^. As a consequence of the rapid depletion of NAD^+^ induced by NQO1 activation, the enzymatic activity of SIRT1, which requires NAD^+^ as a substrate to receive the acetyl group, was found significantly decreased (Figure 3H). SIRT1 is an important deacetylase that is involved in the deacetylation of a growing number of both histone and non-histone substrates including p53 and FOXO1. Because SIRT1 is well known to be an important sensor of redox condition, the downregulation and decreased activity of SIRT1 may be explained by NQO1 substrates triggered excessive oxidative stress which may surpass the adaptive response of SIRT1 anti-oxidative signaling. We and others have previously found that NQO1 activation induced cell death is largely p53-independent. Herein, we validated that FOXO1 determinates NQO1 activation induced apoptotic cell death (Figure 2). Because of the decreased SIRT1 activity, the acetylated FOXO1 is accumulated and translocated to the nuclear to transcriptionally activate apoptotic signaling. Notably, NAD^+^-SIRT1-FOXO1 pathway was found also disrupted in A549 tumor xenografts treated with NQO1 substrates (Figure 6). More importantly, the de-novo NAD^+^ synthesis pathway and SIRT1 activity were found significantly enhanced in the tumor tissues of human NSCLC (Figure 7). Previous studies also demonstrated that SIRT1 is overexpressed in some cancers and has an obvious correlation with poor prognosis of patients by promoting tumor metastasis [36]. These findings indicate that cancer cells may require enhanced NAD^+^ synthesis and SIRT1 activity for survival and promotion. NQO1 activation results in rapid depletion of NAD^+^ and the repression of SIRT1 activity, offering a reasonable rationale to develop NQO1 bioactive agents for the therapy of NSCLC. Moreover, since NQO1 and multiple keynotes involved in de-novo NAD^+^ synthesis (such as LAT1and IDO) are overexpressed in a number of cancers, our study suggests that the combinatory therapy with NQO1 activating agents and the inhibitor of de-novo NAD^+^ synthesis may be a promising strategy for the therapy of NSCLC and other cancers.

**Figure 8:**
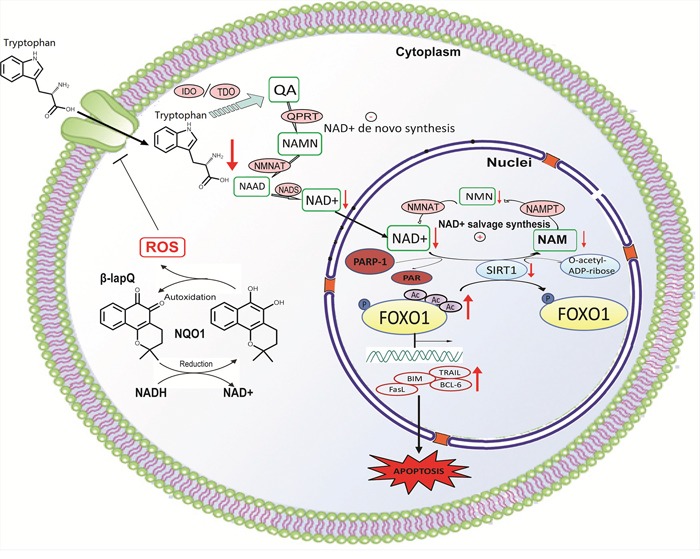
Mechanistic link from NQO1 activation, de-novo NAD^+^ synthesis to SIRT1-FOXO1 signaling NQO1 activation induces rapid downregulation of LAT1, resulting in decreased de-novo NAD^+^ synthesis, together with PARP-1 hyperactivation to induce a rapid depletion of intracellular NAD^+^. Because SIRT1 relies on NAD^+^ for the deacetylation reaction, the reduced NAD^+^ results in the repression of SIRT1 activity, leading to the increased accumulation of acetylated FOXO1 which translocates to the nuclear to transcriptionally activate apoptotic signaling.

In summary, our study develops a mechanistic link from LAT1 mediated uptake of tryptophan and subsequent de-novo NAD^+^ synthesis to SIRT1-FOXO1 regulated cellular apoptosis (Figure 8). By this mechanistic link, our study provides new insights to better understanding the role of tryptophan depletion and de-novo NAD^+^ synthesis in cancer biology and therapy, and may pave the way to new therapeutic options in NSCLC via dual targeting NQO1 and the components in de-novo NAD^+^ synthesis.

## MATERIALS AND METHODS

### Cell lines and transfections

Human NSCLC cell lines A549 and H596 were purchased from the American Type Culture Collection (ATCC, USA) and isogenically matched NQO1-expressing H596 cells were generated by infecting with lentiviral vectors expressing NQO1 or empty vector. LAT1 plasmid was kindly provided by Prof. Chen L from Tsinghua University (Beijing, China). Cells were transfected with FOXO1, NQO1 siRNA, SIRT1 siRNA or LAT1 siRNA (Invitrogen) using lipofectamine™ RNAiMAX transfection reagent (Invitrogen) according to the manufacturer's reverse transcription protocol. FOXO1, NQO1 and LAT1 siRNA sequences are shown in Supplementary Table S1.

### Specimens

Primary specimens and their matched normal adjacent tissues from six patients with locally advanced NSCLC were collected and snap-frozen at the time of surgery. None of the patients had received any chemotherapy or radiotherapy before surgical resection. The study was conducted according to the Helsinki Declaration. The tissues were taken only for patient who agreed to take the exam for the purpose of laboratory research.

### Determination of cytotoxicity and apoptosis

In MTT assay, 20 μL of MTT (3-(4,5-dimethylthiazol-2-yl)-2,5-diphenyl tetrazolium bromide) was added to each well in the 96-well plates and incubated for 4 h at 37°C. MTT solution was removed and 150 μL of DMSO was added to dissolve MTT crystals. Quantitation was performed using a microplate reader with 490 nm wavelength after agitating the plates for 10 min on a shaker.

Apoptosis was quantified by using APO-BRDU™ Kit (BD Biosciences, USA) or AnnexinV-FITC/PI Apoptosis Detection Kit (BD Biosciences, USA). Cells were harvested and stained according to the manufacturer's instruction. Labeled cells were analyzed with flow cytometry FACS Calibur (BD Biosciences, USA).

### Semi-quantitative RT-PCR and western blot analysis

Total RNA was isolated using TRIzol (Invitrogen) and reverse transcribed according to the manufacturer's protocol (Takara). qRT-PCR was performed with SYBR Premix ExTaq™ II (Takara) in a reaction volume of 10 μL. PCR conditions were 95°C for 1 min, followed by 40 cycles of 95°C for 5 seconds, 60°C for 30 seconds, and 72°C for 30 seconds. β-actin gene was used as an endogenous control. Design of primers is shown in Supplementary Table S2.

For western blotting, cells were extracted in lysis buffer, resolved by SDS-PAGE and transferred to nitrocellulose membranes. Proteins were detected using specific primary antibodies directed against SIRT1, Ac-FOXO1, FOXO1, NQO1, LAT1, PARP-1, TRAIL, Bim, FasL or BCL-6 (Supplementary Table S3). The protein expression levels were normalized with GAPDH. After washing with TBST, the membrane was incubated with HRP-conjugated secondary antibody for 1 h. The signal was detected by enhanced cheniluminescence (ECL, Millipore).

### Immunocytochemistry and immunofluorescence

For immunocytochemical staining of FOXO1, cells were fixed in 4 % paraformaldehyde and permeabilized by 0.2 % Triton X-100, blocked in 3 % BSA and incubated overnight at 4°C with primary antibodies for FOXO1 or LAT1. FITC-conjugated secondary antibody was incubated after FOXO1 staining at 37°C for 1 h. Alexa Fluor^®^ 594 secondary antibody was incubated after LAT1 staining at 37°C for 1 h. All cells were stained with DAPI for nuclear observation. Cells were then visualized by confocal fluorescence microscopy.

Hoechst staining was used to evaluate DNA damage. Cells were fixed with 4 % paraformaldehyde in PBS for 1 h at 4°C and stained with 0.5 μg/mL of Hoechst 33342 in the dark for 30 min. Images were analyzed on a fluorescence microscope.

### SIRT1 activity assay

SIRT1 activity was measured using a fluorometric SIRT1 assay kit (Sigma CS1040) according to the manufacturer's instructions. In brief, 20 μL of whole cell extracts prepared with lysis buffer were added to a black side-clear bottom 96-well plate and incubated with 10 μL of fluorometric SIRT1 substrate and 5 μL of NAD^+^ solution for 30 min at room temperature, and the reactions were stopped by adding 5 μL of developer solution and incubating for 10 min. Experimental values are represented as a percentage of the control. Filter excitation and emission was set at 340 nm and 430 nm wavelengths, respectively.

### NAD^+^ and NAD^+^ synthetic pathway intermediates analysis

Cells were precipitated with 80 % methanol containing 50 ng/ml of 2-chloroadenosine in −80°C for 20 min and were scraped and transferred in to EP tube, oscillated at 1400 r for 5 min, then centrifuged (14,000 g, 5 min at 4°C) and evaporated to dryness. Cells were resuspended with 100 μl of ultrapure water and centrifugation (18,000 g, 10 min, 4°C) twice.

NAD^+^ and several NAD^+^ synthetic pathway intermediates including NAM, NAAD, NMN, NAMN, NADH, 3-HK, KYN, and Trp, as well as ^15^N2 labeled Trp, ^15^N labeled NAD, ^15^N labeled NADH were identified and quantified by a Shimazu Ultra Performace LC system (Shimazu Corporation, Kyoto, Japan) interfaced to an API 4000+ triple quadrupole mass spectrometer (Applied Biosystems, Forster City, CA, USA) outfitted with a turbo ion-spray ionization source. The mass spectrometer was operating at the following parameters: ionspray voltage, 5.0 kV; source temperature, 550°C; curtain gas, 20; CAD gas, 9; nebulizer gas (GS1), 55; auxiliary gas (GS2), 50.

Analytes were separated using an amide-column (3 x100 mm i.d., 5 μm, Chrom-Matrix Inc) maintained at 40°C. Mobile phase was water containing 5 mM ammonium acetate (A) and methanol (B). A gradient from 80 % to 20 % B in 4 min, maintained for 3.5 min and then to 80 % B from 7.5 to 15 min was applied at a flow rate of 0.2 ml/min. Injection volume was 10 μl. The multiple reaction monitoring (MRM) and parameters performed for the determination of the metabolites were listed in Supplementary Table S4. The MRM mode dwell time was 50 ms. Analyst version 1.5.2 software (Applied Biosystems) was used for data acquisition and processing.

### Tumor xenograft study

All animal experiments were approved by the ethics committee of China Pharmaceutical University. A549 cells seeded nude mice were provided by SLAC Laboratory Animal (Shanghai, China). Tumor sizes were regularly measured using calipers, and volumes were calculated using the following formula: volume (mm^3^) =length*width*width/2. Animals were initially randomized into 3 groups according to the tumor sizes (6 mice/group). Groups were set as A, vehicle; B, β-lap (20 mg/kg); C, Tanshinone IIA (30 mg/kg). Treatments were started when average tumor volumes reached 100 mm^3^. Agents were administered intraperitoneally every day for 2 weeks. Tumor sizes were monitored and measured every other day. Differences in tumor volumes were analyzed statistically by comparing the slopes of the regression lines for plots of tumor volume vs. days of drug treatment in mice receiving different treatments. Tumors were collected and prepared for RT-PCR assays, western blot assays and NAD^+^ detection after mice were sacrificed.

### Statistical analysis

Data are presented as mean ± SEM. Student's t-test or Mann–Whitney tests were performed using Prism software. Statistical significances in t-test are plotted as following: *P<0.05 and **P<0.01.

## SUPPLEMENTARY FIGURES AND TABLES


