# Supplementary material for: De-novo NAD+ synthesis regulates SIRT1-FOXO1 apoptotic pathway in response to NQO1 substrates in lung cancer cells
Source: Oncotarget. 2016 Aug 23;7(38):62503–19. doi: 10.18632/oncotarget.11526 (PMC5308742; doi:10.18632/oncotarget.11526)
Supplement: Supplementary file 1 [file oncotarget-07-62503-s001.pdf]

De-novo NAD$^+$ synthesis regulates SIRT1-FOXO1 apoptotic pathway in response to NQO1 substrates in lung cancer cells

SUPPLEMENTARY FIGURES AND TABLES

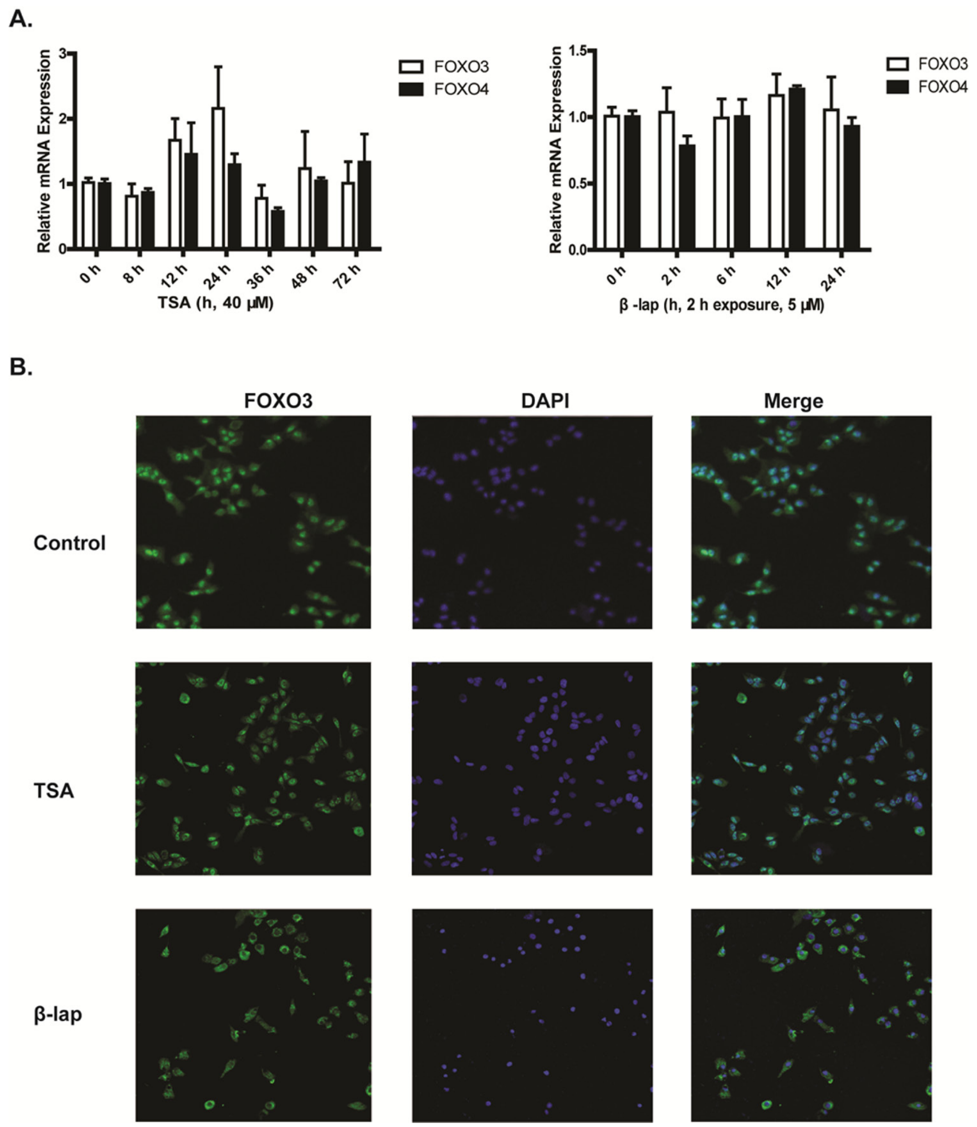

Supplementary Figure S1: NQO1 substrates have no significant effect on FOXO3 and FOXO4 activation. A. FOXO3 or FOXO4 mRNA levels. **B.** FOXO3 nuclear translocation.

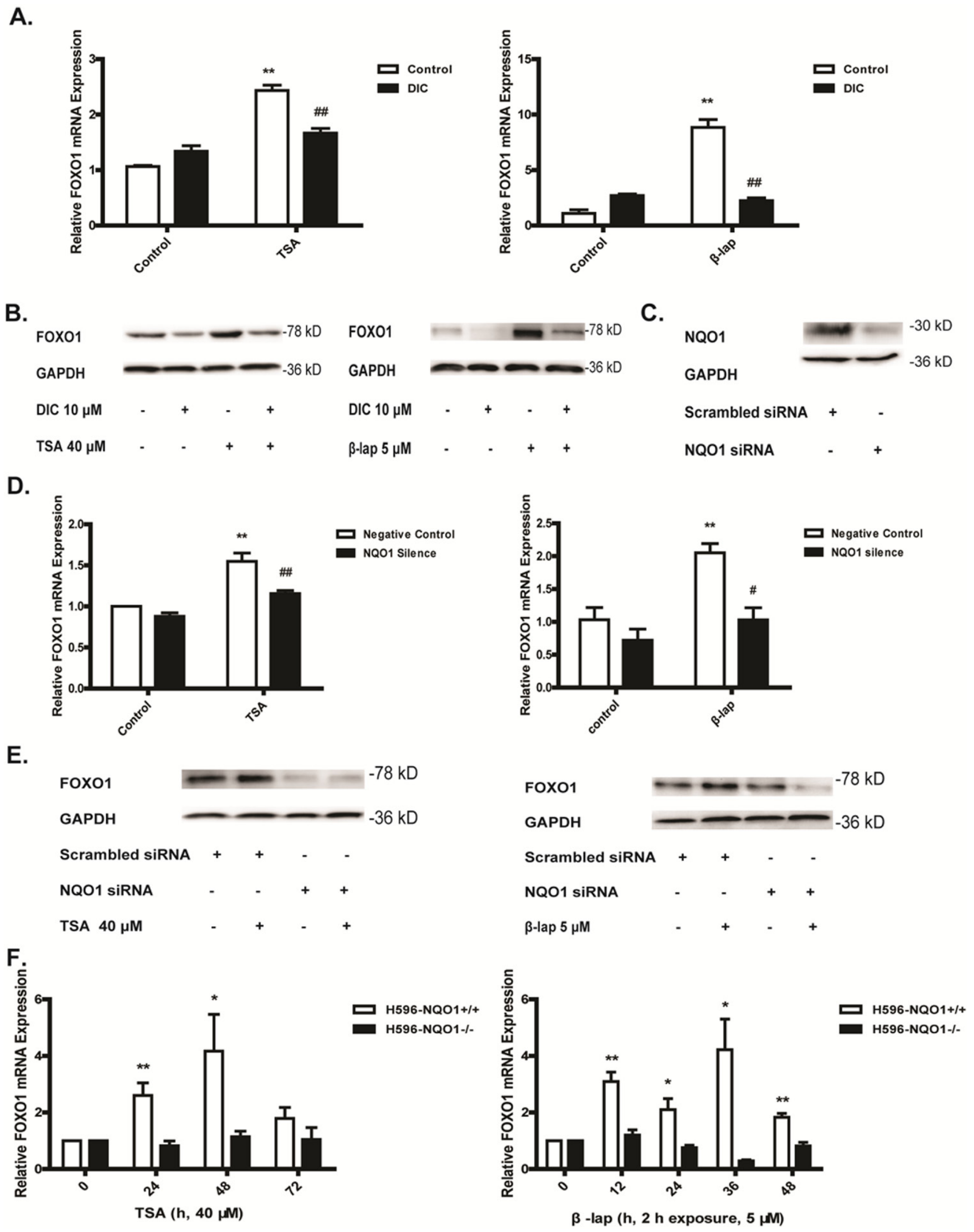

Supplementary Figure S2: Activation of FOXO1 by TSA or β-lap is NQO1-dependent. A. FOXO1 mRNA levels. **B.** FOXO1 protein levels. **C.** NQO1 silencing efficacy **D.** FOXO1 mRNA levels. **E.** FOXO1 protein levels. **F.** FOXO1 mRNA levels in H596-NQO1$^{+/+}$ cells/H596-NQO1$^{-/-}$ cells. Data are shown as mean ± SEM of three independent experiments, *P<0.05, **P<0.01 VS control; #P<0.05, #P<0.01, DIC or NQO1 siRNA pretreatment VS corresponding control, Student's *t* test.

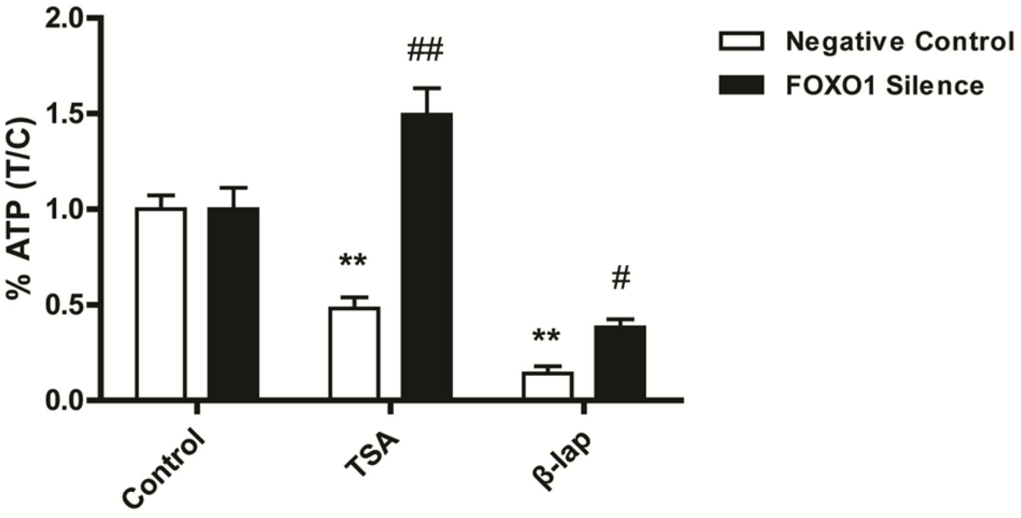

Supplementary Figure S3: FOXO1 silencing reverses TSA and β-lap induced decrease of ATP. The amount of ATP was determined with Molecular Probes' ATP Determination Kit and relative ATP % was calculated with untreated cells as negative control. Data are shown as mean ± SEM of three independent experiments, *P<0.05, **P<0.01 VS control; #P<0.05, #P<0.01, DIC or NQO1 siRNA pretreatment VS corresponding control, Student's *t* test.

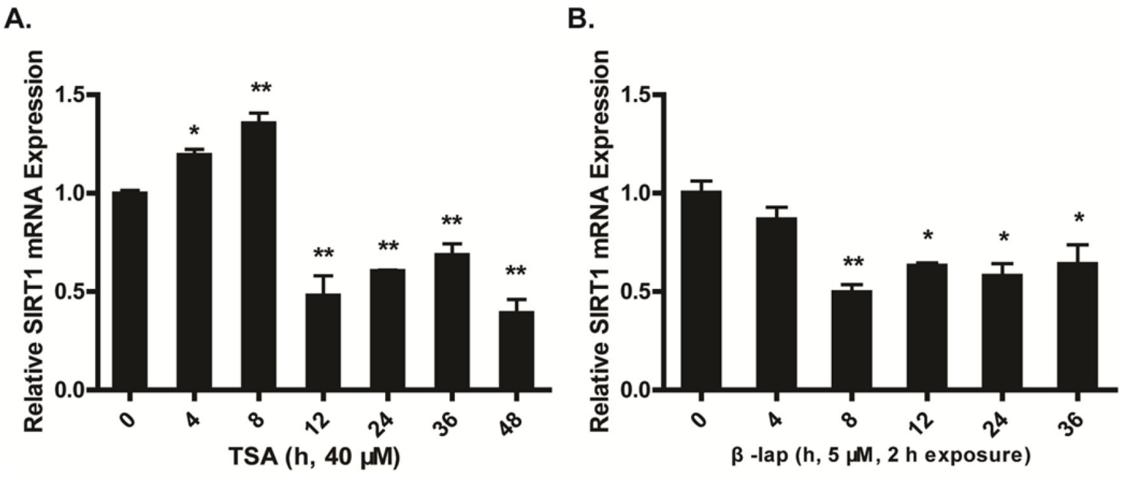

Supplementary Figure S4: NQO1 substrates reduce SIRT1 mRNA level. A. SIRT1 mRNA level during time course of TSA exposure. **B.** SIRT1 mRNA level during time course of β-lap exposure. Data are shown as mean ± SEM of three independent experiments, *P<0.05, **P<0.01 VS control, Student's *t* test.

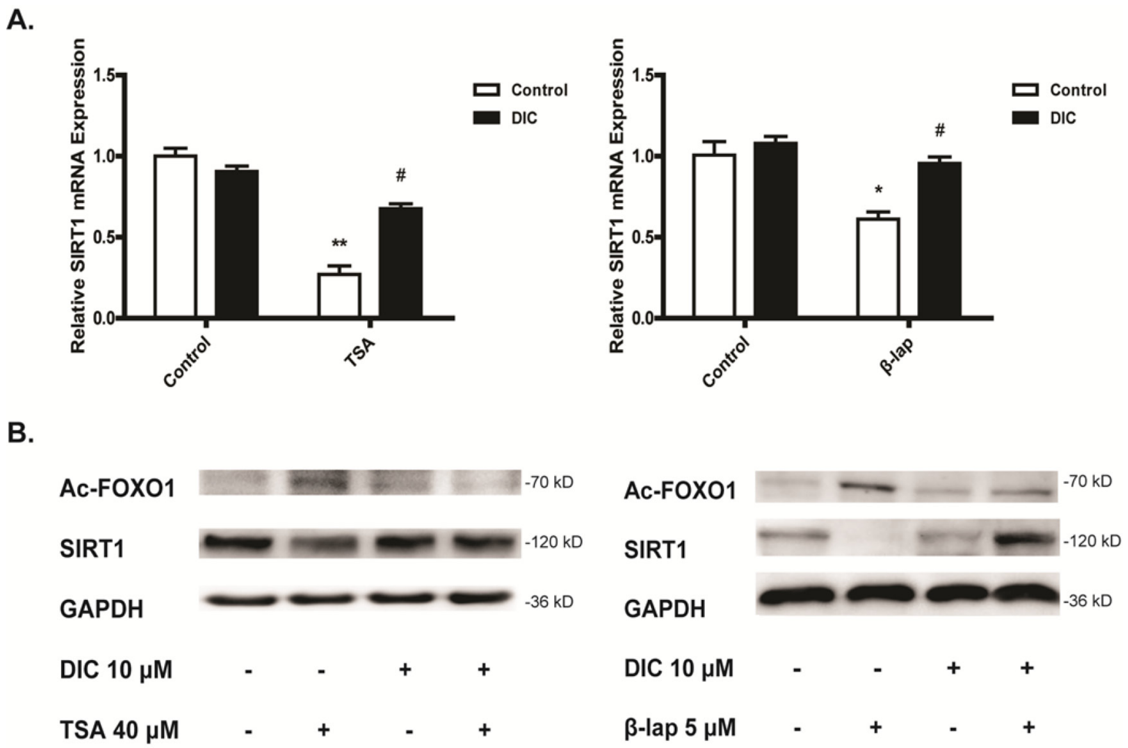

Supplementary Figure S5: TSA or β-lap induced SIRT1 repression is NQO1-dependent. A. SIRT1 mRNA levels. **B.** SIRT1 and Ac-FOXO1 protein levels. Data are shown as mean ± SEM of three independent experiments, *P<0.05, **P<0.01 VS control; #P<0.05, DIC pretreatment VS corresponding control, Student's *t* test.

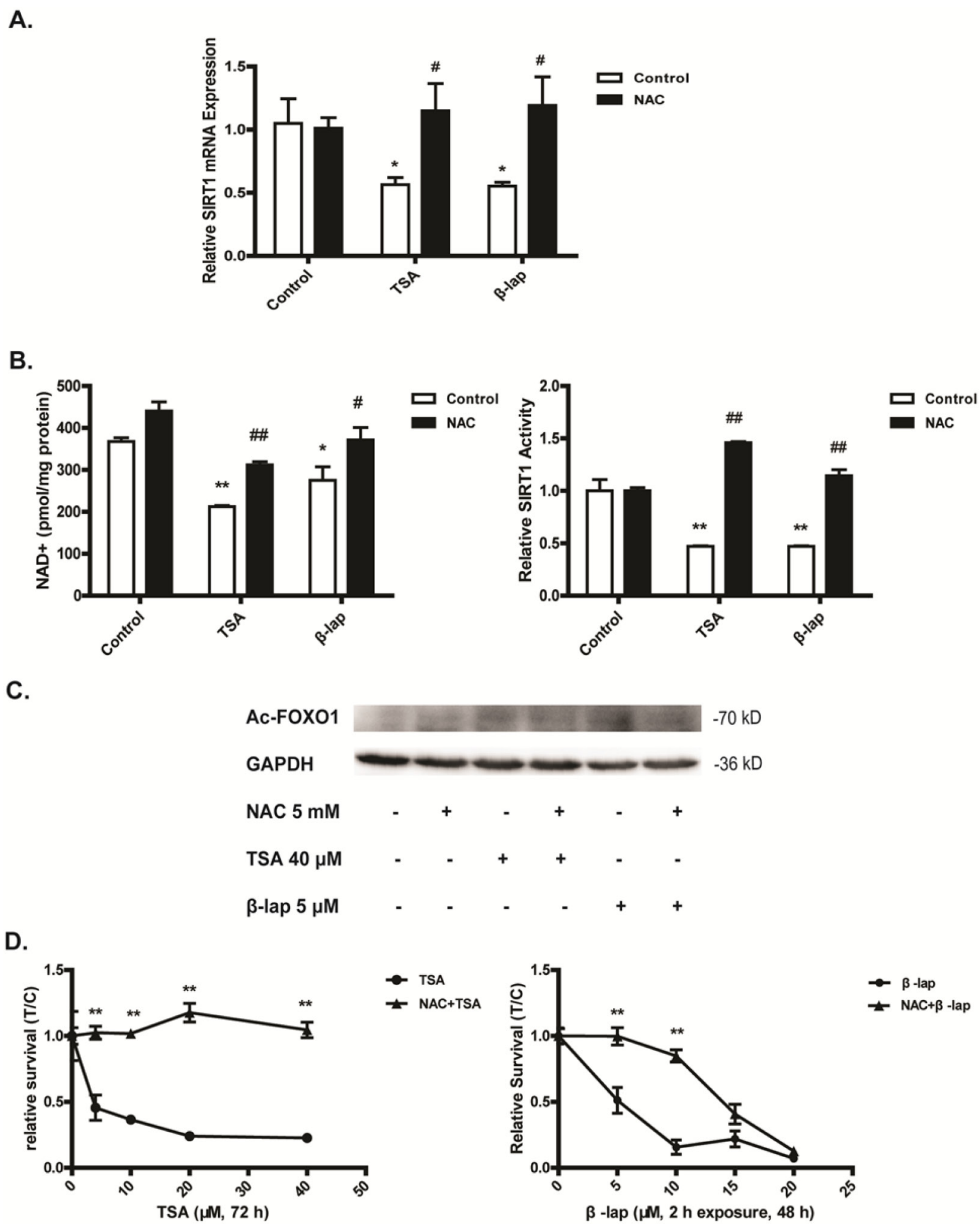

Supplementary Figure S6: NAC combats against the effects of NQO1 activation. A. SIRT1 mRNA levels. **B.** NAD^+ levels and SIRT1 activity. **C.** PARP-1 and Ac-FOXO1 protein expression. **D.** Cytotoxicity. Data are shown as mean ± SEM of three independent experiments, *P<0.05, ** P<0.01 VS control; #P<0.05, ##P<0.01, NAC pretreatment VS corresponding control, Student's *t* test.

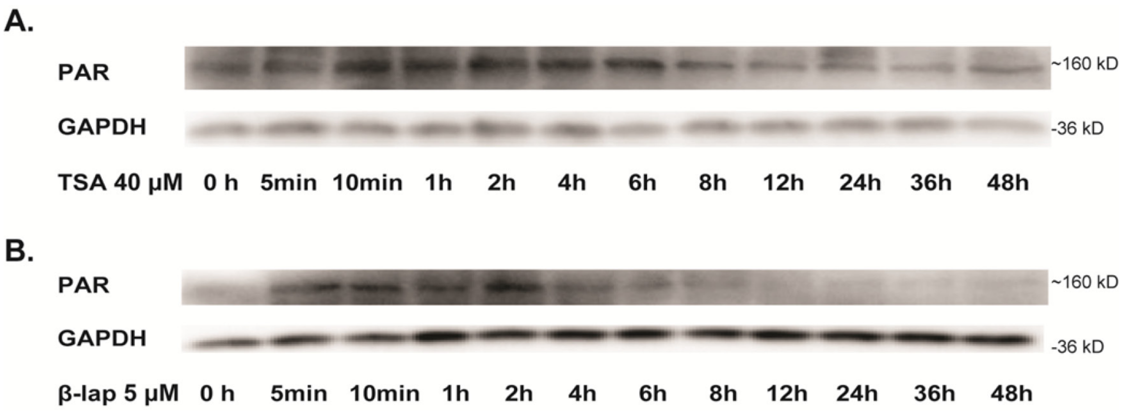

Supplementary Figure S7: NQO1 substrates induce PARP-1 activation. PAR polymer formation after TSA **A.** or β-lap **B.** Data are shown as mean ± SEM of three independent experiments, *P<0.05, ** P<0.01 VS control, Student's *t* test.

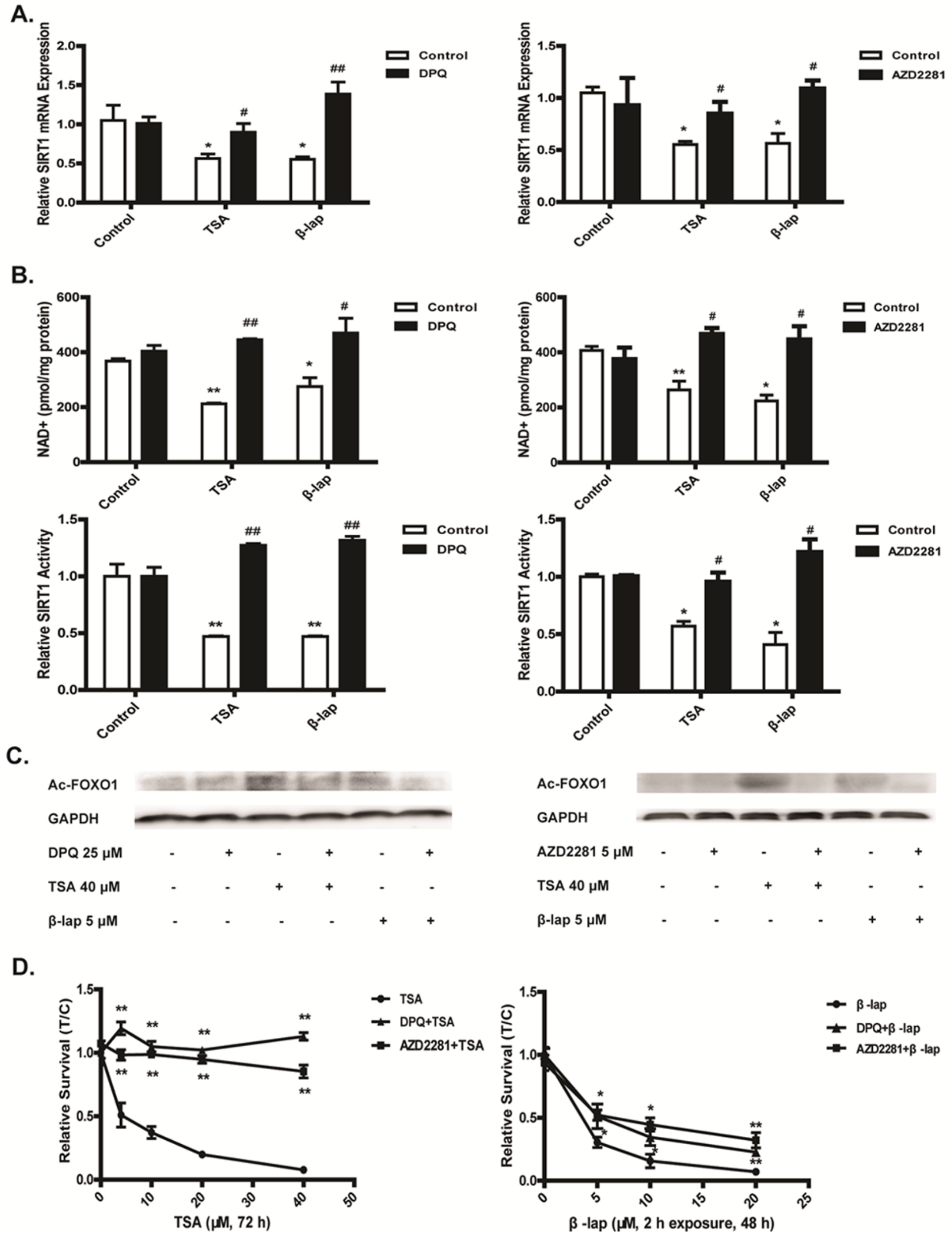

Supplementary Figure S8: PARP-1 inhibitor DPQ reverses TSA or β-lap induced cell death. **A.** SIRT1 mRNA. **B.** NAD^+ levels and SIRT1 activity. **C.** Ac-FOXO1 accumulation. **D.** Cytotoxicity. Data are shown as mean ± SEM of three independent experiments, *P<0.05, ** P<0.01 VS control; #P<0.05, ##P<0.01, DPQ pretreatment VS corresponding control group, Student's *t* test.

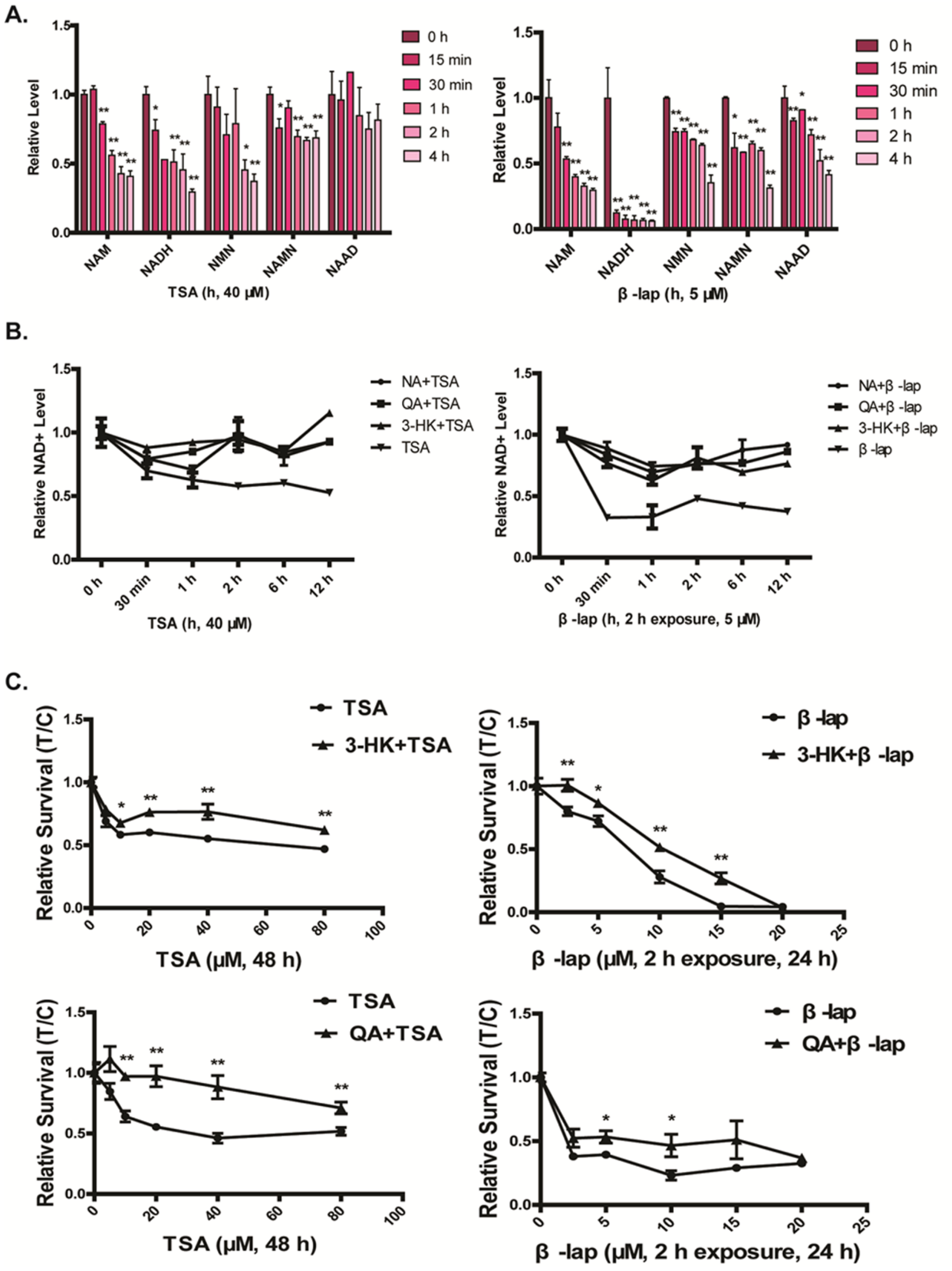

Supplementary Figure S9: De-novo NAD$^+$ synthesis regulates NQO1 activation induced cytotoxicity. A. Levels of the major intermediates involved in NAD$^+$ synthesis detected by LC-MS^n. **B.** NAD$^+$ levels. **C.** Cytotoxicity. Data are shown as mean ± SEM of three independent experiments, *P<0.05, **P<0.01 VS control, Student's *t* test.

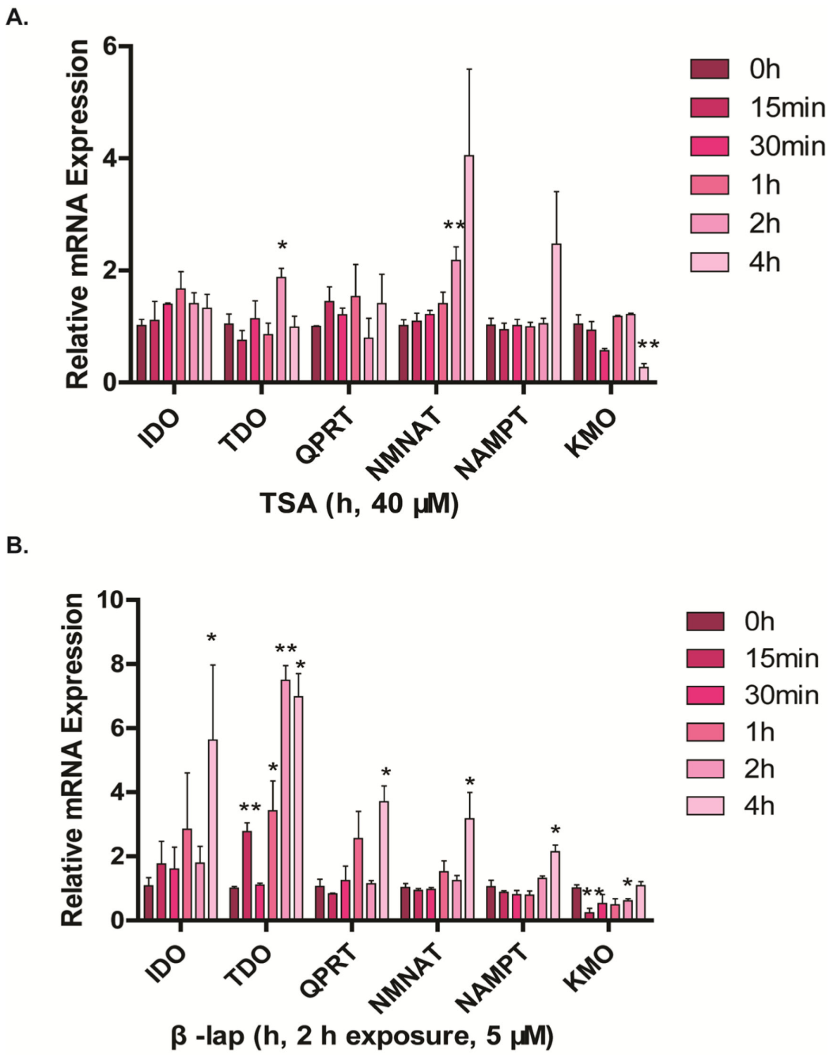

Supplementary Figure S10: NQO1 substrates compensatively up-regulate NAD$^+$ synthetic enzymes. A. NAD$^+$ synthetic enzymes mRNA levels after TSA exposure. **B.** NAD$^+$ synthetic enzymes mRNA levels after β-lap. Data are shown as mean ± SEM of three independent experiments, *P<0.05, ** P<0.01 VS control, Student's *t* test.

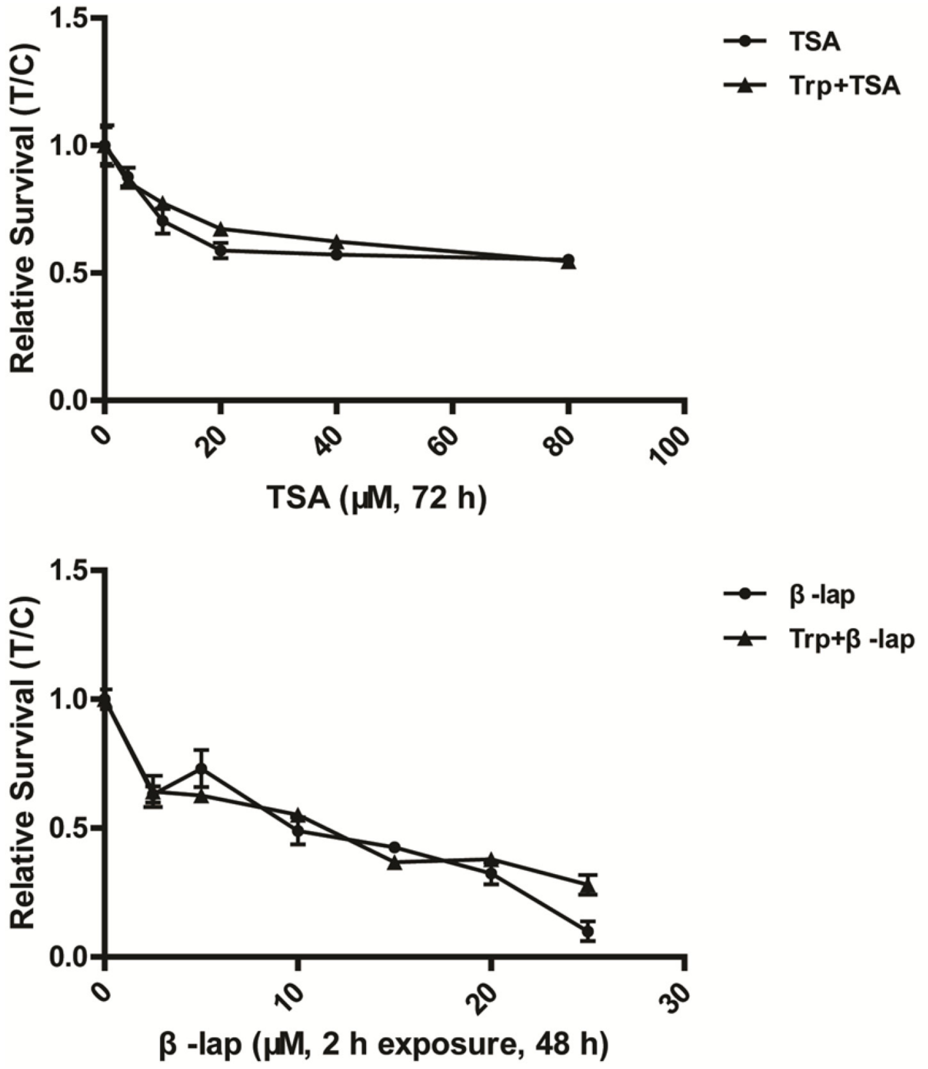

Supplementary Figure S11: Tryptophan affects little on NQO1 substrates cytotoxicity. NQO1 substrates cytotoxicity with or without tryptophan pretreatment.

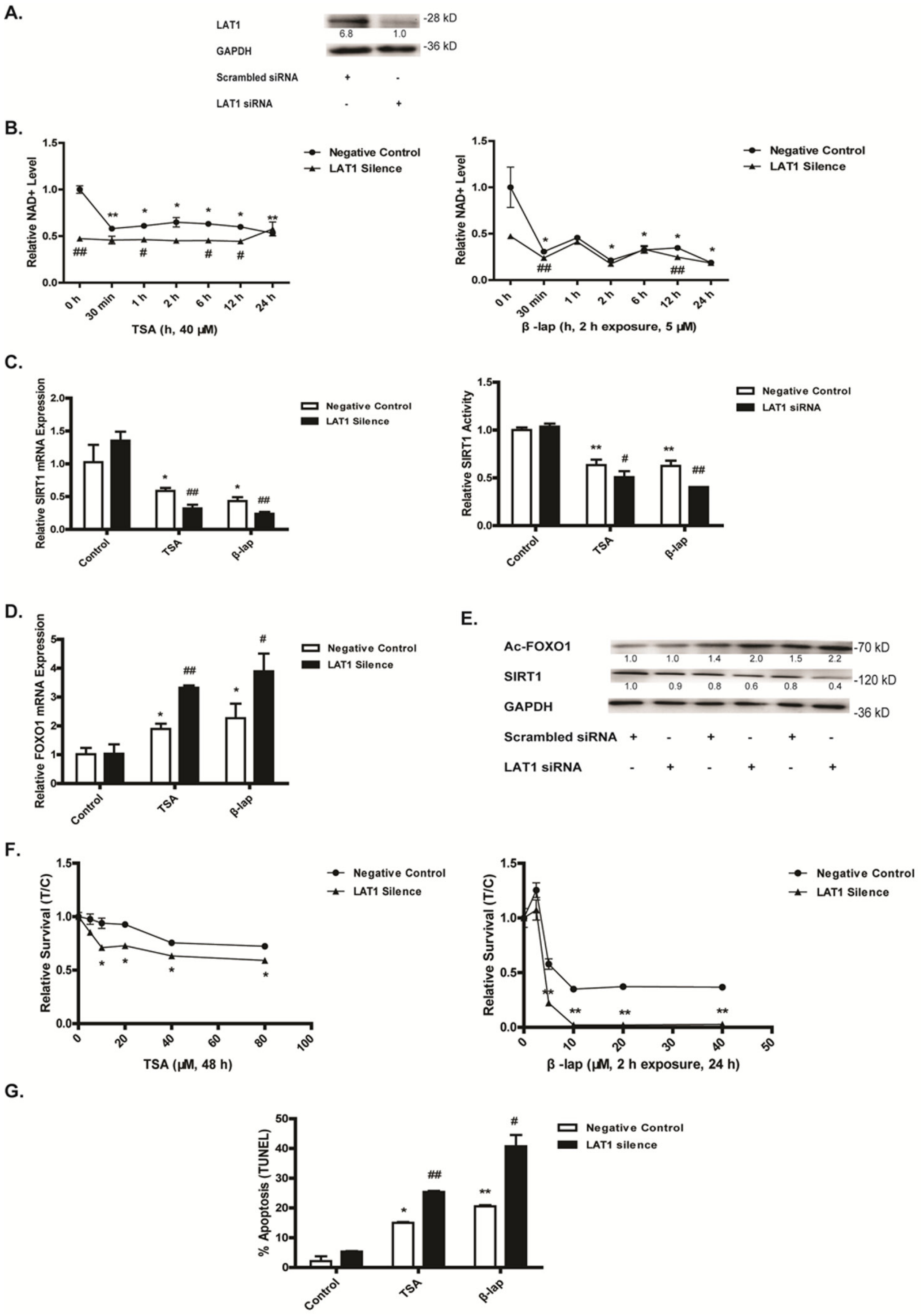

Supplementary Figure S12: LAT1 silencing increases NQO1 activation induced cell death. Cells were pretreated with LAT1 siRNA for 24 h and the efficacy was evaluated by western blot **A.** The NAD^+ level **B.** Cells were then treated with TSA (40 µM, 24 h) or β-lap (5 µM, 2 h withdraw, 12 h). The mRNA level and enzyme activity of SIRT1 **C.** The mRNA level of FOXO1 **D.** The protein levels of Ac-FOXO1 and SIRT1 **E.** Cytotoxicity **F.** and apoptosis test **G.** were performed after cells treated with TSA (40 µM, 48 h) or β-lap (5 µM, 2 h withdraw, 24 h). Data are shown as mean ± SEM of three independent experiments (*P<0.05, **P<0.01, TSA or β-lap treatment compared with control cells; #P<0.05, ##P<0.01, LAT1 siRNA treatment compared with scrambled siRNA treatment).

Supplementary Table S1: siRNA sequences

siRNA	siRNA sequences
FOXO1 siRNA	AGUCUAAGCGCUCAAUGAACAUGCC;<br>GGCAUGUUCAUUGAGCGCUUAGACU
NQO1 siRNA	AAAUGAUGGGAUUGAAGUUCAUGGC;<br>GCCAUGAACUUCAAUCCCAUCAUU
SIRT1 siRNA	UACAAAUCAGGCAAGAUGCUGUUGC;<br>GCAACAGCAUCUUGCCUGAUUUGUA
LAT1 siRNA	CACAGACUGCCAGGCUCCUACGACA,<br>UGUCGUAGGAGCCUGGCAGUCUGUG

Supplementary Table S2: Primer sequences for qRT-PCR

Gene	Primer sequences
FOXO1	TCATGTCAACCTATGGCAG;<br>CATGGTGCTTACCGTGTG;
FOXO3	CATCATGGCAAGCACAGAGT;<br>CAGGTCGTCCATGAGGTTTT;
FOXO4	CAGCCAGTTCATCAAGGTTCAC;<br>CCACATATCCGCTTCTTCACG;
SIRT1	TCAGTGTCATGGTTCCTTTGC;<br>AATCTGCTCCTTTGCCACTCT;
PARP-1	GGCACTCTTGGAGACCATGTCA;<br>AAGGCGAATGCCAGCGTTAC
CD38	GCTAAAACAACCACAGCGACTGG;<br>ACCCCGCCTGGAGCCCTA TG
NAMPT	AAGAGACTGCTGGCATAGGA;<br>ACCACAGATACAGGCACTGA;
QPRT	CACGTGGCAGGCACGAGGAGG;<br>GAGGGAGAAATCAAGGGCTGG;
IDO	TCACAGACCACAAGTCACAGC;<br>AGTTGGCAGTAAGGAACAGCA;
TDO	CTTAGTAAAGGTGAAAGACGG;<br>GTCCATAAGAGAAGTCAGCA;
KMO	AGAGATGCGAGCACATGTCAA;<br>CCATGGTCTTCTCAAGCGGA
y+LAT1	GAAGGAGGAGCATCAGACCA;<br>CCCAGTTCCGCATAACAAAG;
ATA2	AACTACTCCTACCCCACCAAG;<br>TAAGGTGGTGTTTATTGTTTC;
homo-ACTB	AAGAGCTACGAGCTGCCTGAC;<br>TCCTGCTTGCTGATCCACAT

Supplementary Table S3: Details of antibodies used in this study

Name of Antibody	Manufacturer	Cat. No.
SIRT1	Santa Cruz Biotechnology	sc-15404
FOXO1	Cell Signaling Technology	2880
FOXO3	Cell Signaling Technology	2497
Ac-FOXO1	Santa Cruz Biotechnology	sc-49437
NQO1	epitomics	S2173
LAT1	Abcam	ab32070
PARP-1	Cell Signaling Technology	9542
TRAIL	Cell Signaling Technology	3219
BIM	Cell Signaling Technology	2933
FasL	BD pharmingen™	556374
BCL-6	Santa Cruz Biotechnology	sc-858
GAPDH	Shengxing	SAP1646
Anti-rabbit IgG, HRP-linked Antibody	Cell Signaling Technology	7074
Anti-mouse IgG, HRP-linked Antibody	Cell Signaling Technology	7076
Goat anti-Rabbit IgG (H+L) Secondary Antibody, FITC conjugate	Life Technologies	65-6111
Anti-rabbit IgG Alexa Fluor® 594 Conjugate	Life Technologies	R37117

Supplementary Table S4: Parameters of NAD^+ and NAD^+ synthetic intermediates in MRM detection with LC-MS

Compound	m/z of precursor ion	m/z of product ion	DP	CE
NAD^+	664.3	136.3	95	60
NAAD	665.2	665.2	87	5
NMN	335.2	123.2	47	21
NAMN	336.1	124.0	51	20
NAM	123.2	80.2	55	28
NADH	666.3	649.2	93	23
TRP	205.2	90.9	44	53
$^{15}N2$ labeled TRP	207.2	91.9	44	53
^{15}N labeled NAD^+	665.3	136.3	95	60
^{15}N labeled NADH	667.3	650.2	93	23
2-ClAde	302.2	169.9	70	25
